# Pathophysiology of the Belgrade rat

**DOI:** 10.3389/fphar.2014.00082

**Published:** 2014-04-22

**Authors:** Tania Veuthey, Marianne Wessling-Resnick

**Affiliations:** Department of Genetics and Complex Diseases, Harvard School of Public HealthBoston, MA, USA

**Keywords:** SLC11A2, DMT1, iron, manganese, Belgrade rat

## Abstract

The Belgrade rat is an animal model of divalent metal transporter 1 (DMT1) deficiency. This strain originates from an X-irradiation experiment first reported in 1966. Since then, the Belgrade rat’s pathophysiology has helped to reveal the importance of iron balance and the role of DMT1. This review discusses our current understanding of iron transport homeostasis and summarizes molecular details of DMT1 function. We describe how studies of the Belgrade rat have revealed key roles for DMT1 in iron distribution to red blood cells as well as duodenal iron absorption. The Belgrade rat’s pathology has extended our knowledge of hepatic iron handling, pulmonary and olfactory iron transport as well as brain iron uptake and renal iron handling. For example, relationships between iron and manganese metabolism have been discerned since both are essential metals transported by DMT1. Pathophysiologic features of the Belgrade rat provide us with a unique and interesting animal model to understand iron homeostasis.

## OVERVIEW OF IRON HOMEOSTASIS

Iron is present in hemoproteins, such as the important oxygen carriers hemoglobin and myoglobin, and it is a component of non-heme proteins that carry out other key functions in cellular metabolism, such as mitochondrial aconitase and ribonucleotide reductase. The relevance of iron lies in its ability to cycle reversibly between the ferrous (Fe^2^^+^) and the ferric (Fe^3^^+^) oxidation states (Wessling-Resnick, 1999). Free ferrous iron is a potent catalyst for lipid peroxidation and protein and DNA oxidation since it is able to react with molecular oxygen and generate reactive oxygen species (ROS) through Fenton chemistry (Hentze et al., 2004) Given the biological importance of iron and the potential for its toxicity, iron homeostasis is tightly regulated.

Dietary iron is absorbed through the duodenum. Regulated pathways of iron excretion do not appear to exist, and therefore regulation of iron absorption is critical to maintain iron balance. Iron reaches the liver by portal circulation, where it can be stored until needed or delivered through systemic circulation to peripheral tissues. A significant amount of iron is transported to the bone marrow where erythropoiesis takes place. Senescent red blood cells (RBCs) are phagocytosed by reticuloendothelial macrophages, which catabolize iron from heme to be recycled and used for new RBC synthesis. This recovery is highly efficient so that most of the body iron is usually found in circulating hemoglobin within erythrocytes (Wessling-Resnick, 2000; Hentze et al., 2004).

**FIGURE 1:**
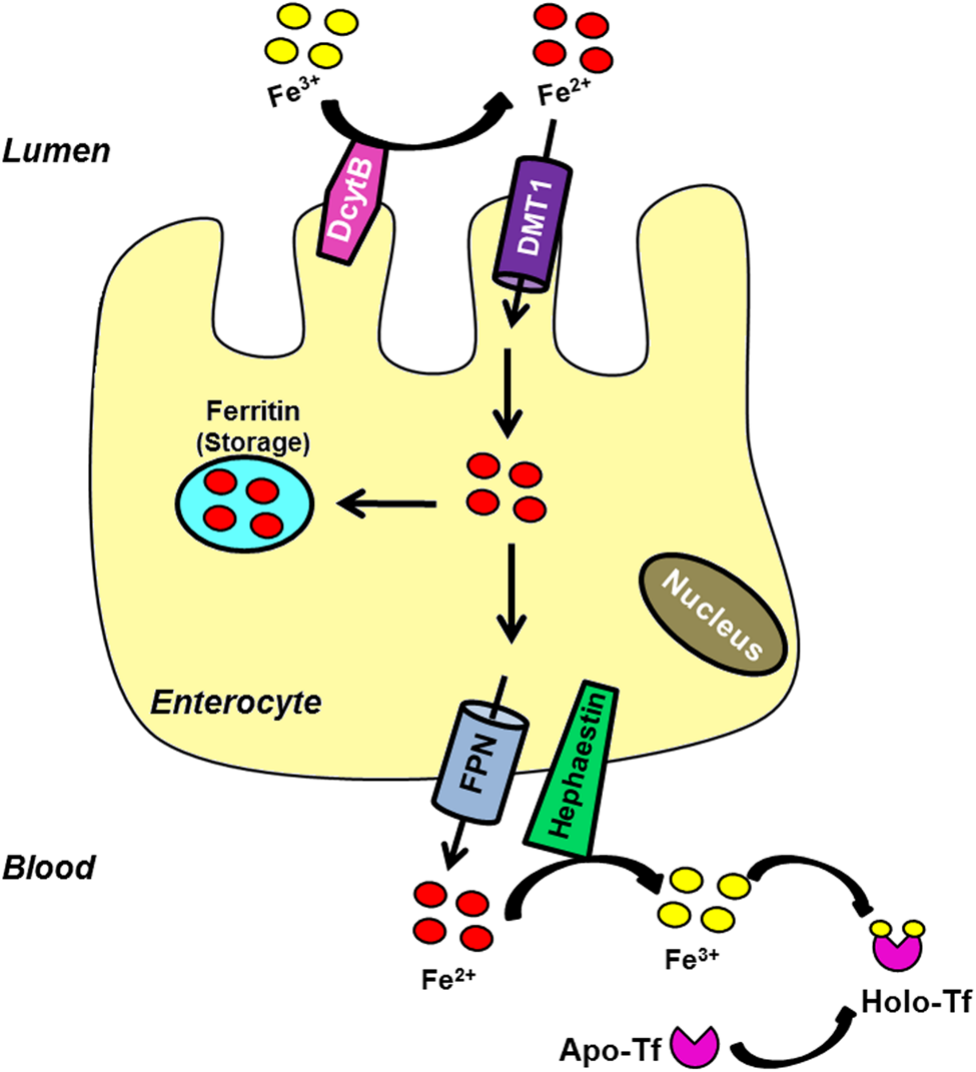
**Intestinal iron uptake.** Luminal ferric iron is reduced (DcytB) at the apical membrane of enterocytes. Ferrous iron is then transported into the cell by the brush border protein DMT1. Once inside the cell, iron can bind to ferritin to be stored or exported across the basolateral surface by FPN. The ferroxidase hephaestin converts it into the ferric form. Ferric iron is bound by apo-Tf to circulate into the blood.

Duodenal absorption of iron begins at the apical membrane of enterocytes, where the reduction of Fe^3^^+^ to Fe^2^^+^ is carried out by DcytB and possibly other ferrireductases (McKie et al., 2001; Mackenzie and Garrick, 2005). Then, iron is transported into the cell through its primary importer DMT1 [divalent metal transporter 1; SLC11A2 (solute carrier family 11, member 2); DCT1 (divalent cation transporter 1)]. Once iron enters the cell it may be stored in ferritin, a protein complex constituted by heavy and light chains that is able to store up to 4500 iron atoms (Harrison and Arosio, 1996). Iron can also be immediately exported from the cell by ferroportin [Fpn; SLC40A1; MTP1 (metal transporter protein 1)], which is located in the basolateral membrane of enterocytes (Abboud and Haile, 2000; Donovan et al., 2000; McKie et al., 2000). The ferroxidase hephaestin mediates its conversion to the ferric state before it is released and bound by serum transferrin (Tf; Wessling-Resnick, 2006). **Figure 1** summarizes the elements involved in this process.

**FIGURE 2:**
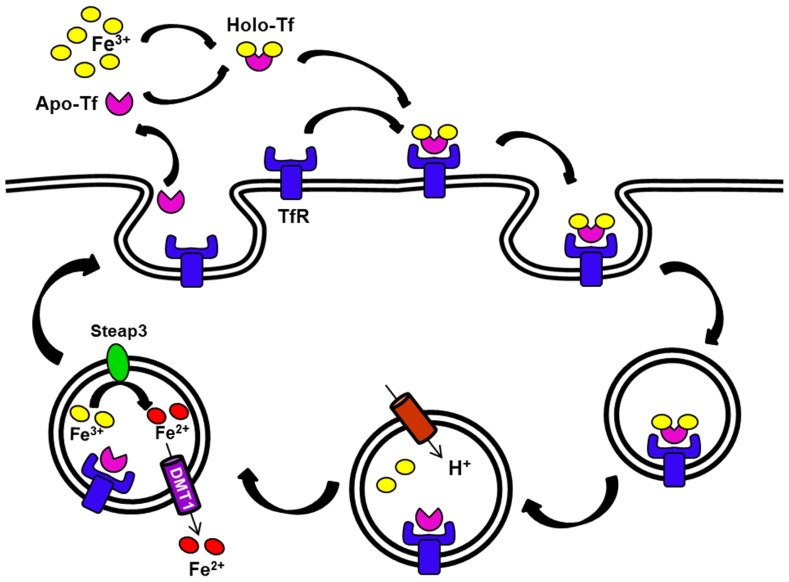
**The transferrin (Tf) cycle.** Apo-Tf binds ferric iron. The resulting Holo-Tf associates with TfR located on the cell surface. The complex TfR–Tf–Fe is then internalized. Iron is released within endocytic vesicles by a process that involves endosomal acidification. Apo-Tf remains bound to its receptor in the endosome and this receptor complex recycles back to the cell surface. Ferric iron is reduced in the endosome (Steap3), and then released into the cytosol by DMT1. The G185R mutation of the Belgrade rat produces DMT1 that is defective in this step, thus some iron remains bound to Tf and recycles back with the receptor.

At the site of liver iron storage, hepatocytes take up iron through two distinct pathways. Tf-bound iron is taken by the Tf cycle (**Figure 2**). In this pathway, iron-loaded Tf binds to its receptor on the cell surface at neutral pH. After binding, the complex is internalized by receptor-mediated endocytosis, entering an endosomal compartment that is acidified by a proton pump. This acidification enables release of iron from Tf and its subsequent transport out of the endosome through DMT1. Subsequently, the apo-Tf–Tf receptor (TfR) complex returns to the cell surface to dissociate at neutral pH (Dautry-Varsat et al., 1983). There are two TfRs that have been characterized: TfR1 is ubiquitously expressed while TfR2 is only expressed in certain tissues, including the liver. Hepatocytes are also capable of importing non-Tf-bound iron (NTBI), which accumulates in circulation during iron loading conditions such as hereditary hemochromatosis. Although DMT1 might be one route for NTBI uptake (Shindo et al., 2006), a member of the SLC39 ZIP family called Zip14 may play a more important role in this process (Liuzzi et al., 2006; Nam et al., 2013; Wang and Knutson, 2013). Once inside of the cell, the iron can be stored as ferritin or released back into the circulation in response to deficiency conditions. The latter process is thought to involve iron export by ferroportin (Wessling-Resnick, 2006).

Because most of the body’s iron is found in hemoglobin, erythrocytes are especially important in iron handling. Erythroid precursors take up iron by the Tf cycle in a highly efficient manner; they probably lack an iron export mechanism since all the iron is retained by the cell, intended for hemoglobin synthesis (Andrews, 2000). The release of iron from the endocytic compartment requires functional DMT1 in these cells (Fleming et al., 1998). A ferrireductase, Steap3, acts to reduce Fe^3^^+^ to Fe^2^^+^, enabling transport to the cytosol (Ohgami et al., 2005). Iron entering the erythron goes to the mitochondria where the major proportion of intracellular iron metabolism takes place (Garrick and Garrick, 2009).

**FIGURE 3:**
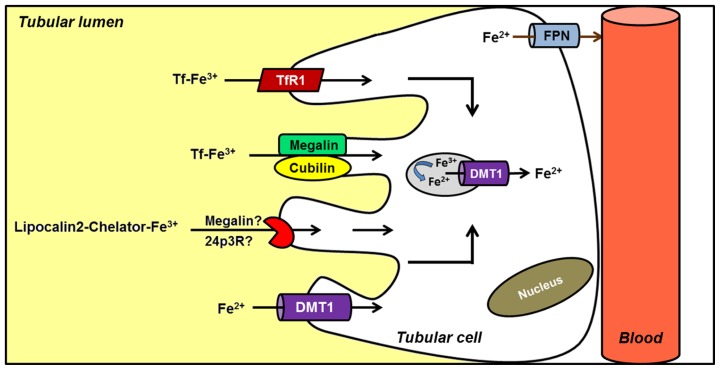
**Hypothetical model of renal iron handling.** Filtered Tf–Fe is reabsorbed in proximal tubule cells by receptor-mediated endocytosis. Potential receptors include cubilin–megalin complex, a possible novel Tf receptor (TfR), and TfR1. Reabsorption of iron bound to a chelator (LCN2) has also been described, although the receptor involved is less clear. Alternatively, ferrous iron is directly imported into the cell by DMT1. Although several features of the model are not completely known, it has been postulated that iron complexes could be endocytosed. Once in the endosome, iron would be reduced and released into the cytosol by endosomal DMT1. Finally, ferrous iron is exported by FPN located in the basolateral membrane; an apical distribution has been noted under conditions of renal failure.

Although iron homeostasis mainly relies on the control of iron efflux from duodenal enterocytes and iron reutilization by macrophages balanced by the demands of heme synthesis by RBCs, increasing data suggest that the kidney could also be involved in systemic iron handling (Moulouel et al., 2013; Veuthey et al., 2013). DMT1 is found at the apical membrane of renal tubular cells (Ferguson et al., 2001; Canonne-Hergaux and Gros, 2002; Veuthey et al., 2008). Wareing et al. (2000) demonstrated that a significant amount of iron is filtered at the glomerulus and the majority is reabsorbed along the nephron. There is evidence to suggest that Tf-bound iron may be filtered and reabsorbed along the proximal tubule following endocytosis via the megalin–cubilin pathway, a possible novel TfR, and/or the TfR1 (Kozyraki et al., 2001; Smith and Thevenod, 2009). These data support the hypothesis that the kidney, in addition to maintaining its self-supply, may also undertake iron reabsorption and thus contribute to general iron homeostasis (**Figure 3**). Furthermore, renal Fpn expression in proximal tubule-specific ferritin heavy chain-knockout mice is decreased, both in mRNA and protein levels (Zarjou et al., 2013). Under normal conditions Fpn was predominantly expressed in the apical membrane of proximal tubules, while after acute kidney injury Fpn was redistributed to the cytosol and basolateral membrane. This recent finding reinforces the hypothesis that kidney may be more extensively involved in iron metabolism than initially proposed.

## DIVALENT METAL TRANSPORTER 1

DMT1, also known as Nramp2, DCT1, and SLC11A2, was identified in 1997 by two groups using different approaches. Gunshin et al. (1997) uncovered the iron transporter by functional expression cloning and Fleming et al. (1997) determined that defects in its gene were responsible for the microcytic anemia phenotype of *mk* mice. As outlined above, it is the major point of iron entry into the body. DMT1 has 12 putative transmembrane (TM) domains. Predicted glycosylation sites in the fourth extracellular loop and a consensus transport motif in the fourth intracellular loop were defined, with both N- and C-termini determined to be topologically situated within the cytoplasm (Gruenheid et al., 1995; Gunshin et al., 1997). Three negatively charged and highly conserved residues in TM 1, 4, and 7 of DMT1 are suggested to be essential for cation transport. In addition, two histidine residues in the TM domain 6 appear critical to normal function (Lam-Yuk-Tseung et al., 2003). DMT1 selectively imports iron in a pH-dependent manner and it has been described to operate as a H+/divalent cation cotransporter (symporter) but also as a H+ uniporter. Another important feature is the voltage-dependent activity of DMT1 gradient (Gunshin et al., 1997).

Transcription of the SLC11A2 genes encoding DMT1 originates four different mRNA transcripts, called 1A/+IRE, 1A/-IRE, 1B/+IRE, and 1B/-IRE. Alternate promoters determine whether the 5′ end of the mRNA will be exon 1A or exon 1B (Hubert and Hentze, 2002). Moreover, variable 3′ processing give rise to two transcripts that differ in the 3′-translated and untranslated regions (UTRs). One of the transcripts is referred as +IRE because it contains an iron-responsive element (IRE) in its 3′-UTR, while the other is called -IRE because it lacks that element (Lee et al., 1998). The four transcripts encode related but distinct proteins. Thus, +IRE and -IRE encode two isoforms that differ in the C-terminus: an 18 amino acid residue found in the +IRE isoforms while -IRE transcripts have a 25 amino acid residue.

The key difference between +IRE and -IRE transcripts is the fact that the presence of the IRE confers post-transcriptional regulation by iron status. Similar to TfR regulation (Galy et al., 2013), decreased iron supply promotes the interactions of the +IRE DMT1 transcript with iron regulatory proteins (IRPs), resulting in the stabilization of the mRNA (Gunshin et al., 2001; Pantopoulos, 2004). This is one mechanism that accounts for the increased duodenal expression of DMT1 seen under iron deficiency, thus promoting iron uptake (Canonne-Hergaux et al., 1999). Another mechanism is conferred by 5′ regions flanking exon 1A, which contain hypoxia response elements (HREs). In the intestine, HREs are recognized by HIF-2α to modulate DMT1 expression under hypoxic conditions (Mastrogiannaki et al., 2009; Shah et al., 2009).

The isoforms also differ in the sites of expression. Whereas the 1B isoform is ubiquitous, the 1A isoform appears to be tissue-specific. Expression of DMT1 1A isoform in duodenal enterocytes has been reported by several authors, with a gradient of expression from the proximal to distal small intestine (Gunshin et al., 1997; Canonne-Hergaux et al., 1999). The kidney is the tissue with second highest expression of the 1A isoform mRNA of DMT1 (Hubert and Hentze, 2002). On the other hand, both +IRE and -IRE isoforms are expressed in many tissues such as liver, lung, brain, and thymus (Gunshin et al., 1997; Hubert and Hentze, 2002).

As the name implies, DMT1 is able to transport a broad spectrum of divalent metals, although its affinity for Fe^2^^+^ is much higher than for other metals (Mackenzie and Hediger, 2004). Manganese, in particular, possesses many physicochemical properties similar to iron, and both metals have been shown to compete in membrane transport processes such as intestinal absorption (Thomson et al., 1971; Rossander-Hulten et al., 1991) and uptake by erythroid cells (Morgan, 1988; Chua et al., 1996). Like iron, manganese is an essential nutrient, and exogenous expression studies (Conrad et al., 2000; Garrick and Dolan, 2002), and more recent molecular studies (Roth and Garrick, 2003; Thompson et al., 2007b; Illing et al., 2012; Kim et al., 2012) have documented a role for DMT1 in manganese uptake as well as iron transport. Unlike iron deficiency, hypomanganesemia is rare. However, manganese loading or manganism can be caused by excess metal exposure resulting in a Parkinson-like disorder. Loading of this metal is not typically associated with ingestion since hepatic first-pass elimination provides an important protective mechanism against potential toxicity. Since intake of airborne Mn^2^^+^ bypasses the biliary excretion route, distribution of metal directly to the brain can occur, promoting neurotoxicity. Several studies have demonstrated a strong relation between high levels of manganese and impaired behavior (Yamada et al., 1986; Tran et al., 2002). The role of DMT1 not only in iron pathophysiology but also in manganese toxicity will be discussed below.

## THE BELGRADE RAT

Belgrade (*b*) rats were described for first time in 1966 as offspring of an X-irradiated albino rat in Belgrade, Yugoslavia (Sladic-Simic et al., 1966). No differences were observed in litters of F1 generation. When the female was bred with a normal male, 11 apparently normal rats were born, four of which died young. Of the seven remaining, two pairs were mated, producing anemic rats in the F2 generation. In subsequent filial generations anemic rats were also seen, suggesting that offspring were heterozygous for a recessive mutation that caused anemia.

The newborn anemic rats were pale, with growth retardation compared to normal littermates. Peripheral blood smears showed marked microcytosis, anisocytosis, and poikilocytosis that increased with age. The mean value for erythrocytes in the peripheral blood was significantly decreased and a progressive decline in hemoglobin values occurred with age (Sladic-Simic et al., 1966). Adult *b/b* rats are distinguished by lower body weight than +/b rats (Thompson et al., 2007a), and characterized by pinkish retinal reflex and white ears, in contrast with the bright red of *+/b* animals (Ivanovic, 1997). Anemia was also accompanied by a decrease in platelets count and leukocytosis (Ivanovic, 1997).

**FIGURE 4:**
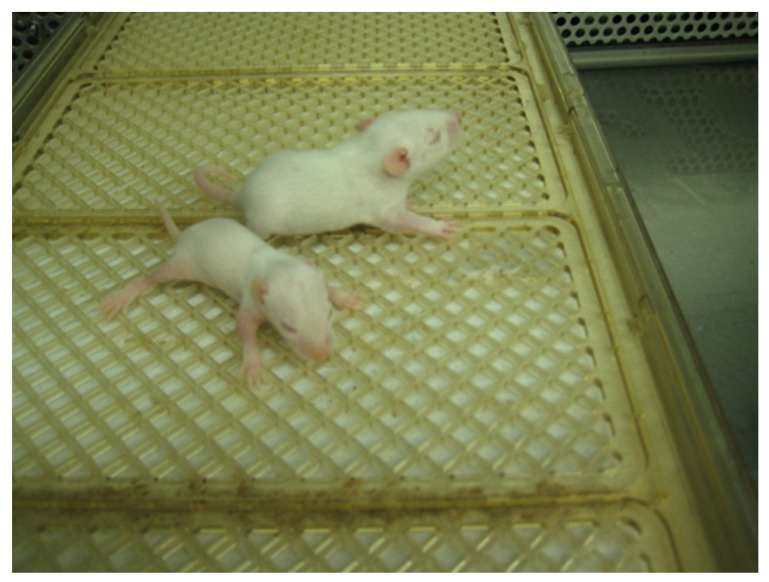
**Belgrade rat.** A *b/b* rat pup (foreground) and heterozygous *+/b* littermate are shown at post-natal day 10. Image courtesy of Dr. Xuming Jia.

In the spring of 1967, a colony of these rats was brought to the U.S. and established in New York (Sladic-Simic et al., 1969). It was maintained by crossing Belgrade males with Wistar female rats. Then, the F1 heterozygous females were back-crossed to homozygous anemic males; some rats from the F2 generation exhibited an anemic condition similar to that described in Belgrade rats. The appearance of the phenotype in F2 generations was compatible with a single autosomal recessive character, as previously demonstrated in Belgrade (Sladic-Simic et al., 1969). This genetic factor was designated as *b* and the homozygous anemic Belgrade rats were called *b/b* (**Figure 4**).

It was nearly 30 years after the discovery of these rats that detailed genetic characteristics of Belgrade rats were ascertained. A glycine-to-arginine substitution (G185R) in the fourth TM domain of DMT1 was reported, resulting in loss of activity of the transporter (Fleming et al., 1998). Remarkably, it was the exact same mutation reported for *mk* mice that led to the identification of DMT1 (Fleming et al., 1997). Despite the fact that increased transcript levels are induced by iron deficiency, relative amounts of protein are reduced (Oates et al., 2000; Yeh et al., 2000; Ferguson et al., 2003), and the function of residual DMT1 may be altered (Yeh et al., 2000; Touret et al., 2004; Xu et al., 2004). Work on the Belgrade rat defined a key role for DMT1 not only in intestinal iron absorption, but also in the Tf iron delivery cycle. In fact, Belgrade rats can display high serum iron due to ineffective erythropoiesis (Thompson et al., 2006).

Since the iron imbalance of *b/b* rats became known, many attempts were made to relieve the anemia, extend lifespan and improve husbandry of the Belgrade rat. Iron supplementation by iron-dextran injections was first tested, but although the anemic state was improved, tissue iron deposition was observed (Garrick et al., 1997). Iron supplementation by diet turned out to be more beneficial since it improved anemia without alteration in red cell morphology. Although in both cases iron treatment raised hemoglobin values, levels remained lower than control (Sladic-Simic et al., 1966). Later on, it was reported that the critical nutritional factor would be the source of iron, given that ferrous iron was more bioavailable than ferric iron (Sladic-Simic et al., 1969; Garrick et al., 1997).

### CHARACTERISTICS OF BELGRADE RAT ERYTHROID CELLS

The complexity of iron metabolism in Belgrade rats triggered many questions about the mechanism underlying the impaired utilization of iron. Since the anemia of *b/b* rats resembled thalassemia in humans, first thoughts were focused on possible differences of hemoglobin characteristics. It was demonstrated that reticulocyte globin synthesis was diminished in *b/b* rats although no major imbalance between α- and β-chain production was observed (Edwards et al., 1978). A lack of sideroblasts was also observed in *b/b* rats, indicating that iron was not accumulated within erythroid cells (Edwards et al., 1978). This finding suggested a defect in iron transport into the erythroid cells rather than a defect in intracellular utilization. To consider the possibility that Belgrade rats harbored a defect in heme synthesis, iron incorporation into heme was inhibited. Iron uptake by reticulocytes became diminished with no evidence of intra-erythrocyte iron accumulation in *b/b* rats compared to controls (Edwards et al., 1978). These data suggested that Belgrade anemia was probably not due to a defect in heme synthesis. The same authors were ultimately able to demonstrate that the main defect related to iron utilization in *b/b* rats lies in a significantly lower reticulocyte iron uptake compared to *+/b* controls.

Each step in the iron uptake mechanism by reticulocytes was studied in detail to decipher the underlying cause of Belgrade anemia (Bowen and Morgan, 1987). When receptor interactions were evaluated, Tf binding affinities between *b/b* and control reticulocytes were found to be similar. The Tf molecule itself had the same molecular weight and net charge compared to Wistar rat Tf (Farcich and Morgan, 1992b). Studies on Tf endocytosis showed a slower mechanism in Belgrade reticulocytes, although the relative decrease was not as great as the defect in iron uptake. In addition, Tf exocytosis rate was similar between Belgrade and control reticulocytes, although *b/b* cells released more iron with Tf than control cells (Bowen and Morgan, 1987). In fact, a significant proportion of the iron taken up by *b/b* reticulocytes returned to the extracellular medium, concordant with reduced iron accumulation. These early studies clearly indicated that Belgrade reticulocytes were defective in iron release after uptake by Tf within endocytic vesicles such that if iron was released, it was unable to pass through the endocytic vesicle membrane to the cytoplasm (Bowen and Morgan, 1987; **Figure 2**).

Garrick et al. (1993b) focused on the Tf cycle to more definitively demonstrate that *b/b* reticulocytes retained only half of iron borne by incoming Tf, while around 90% of iron that entered to normal cells remained intracellular. This evidence confirmed that ineffective iron utilization by *b/b* reticulocytes contributes to the Belgrade defect. Furthermore, when heme synthesis was inhibited, iron from Tf failed to accumulate in the stromal (mitochondrial) fraction or in the non-heme cytosolic fraction (Garrick et al., 1993a). These studies reinforced the idea that the Belgrade defect was related to iron release from Tf or its transport from endocytic vesicles. The significantly lower uptake of iron by erythroid precursors most likely contributes to the increased Tf saturation, serum total iron-binding capacity (TIBC) and serum iron levels reported in *b/b* rats (Thompson et al., 2006). It was also reported that Belgrade reticulocytes have only about half the amount of globin mRNA compared to normal cells, suggesting some sort of a translational defect (Chu et al., 1978). Given that heme regulates mRNA translation in reticulocytes, the failure in this process could be explained because *b/b* reticulocytes contain about 40% of “free” heme compared to *+/b* cells (Garrick et al., 1999b).

Non-Tf-bound iron uptake by erythroid cells was also characterized in Belgrade rats (Garrick et al., 1999a). NTBI uptake differed from Tf–Fe uptake in the pattern of iron distribution between subcellular fractions. Moreover, *b/b* cells only incorporated 20% of the NTBI compared to *+/b* cells, a fraction similar to the residual levels found for Tf–Fe utilization. The results strongly suggested that DMT1 could also play a key role in NTBI acquisition into heme, as well as in iron transport out of endosomes in the reticulocyte Tf cycle. Since the relationship between iron and manganese transport was recognized many years ago, the abnormal iron metabolism seen in erythroid cells of *b/b* rats elicited interest to determine if manganese transport was also affected. Three manganese transport mechanisms have been identified in reticulocytes, one for Mn–Tf and two for the unbound divalent cation Mn^2^^+^, one of low and the other of high affinity (Chua et al., 1996). Impaired manganese uptake from Tf and defective import via the high affinity Mn^2^^+^ transport were both observed in Belgrade reticulocytes. These findings strongly supported the idea that these two pathways utilize the same transporter (Chua and Morgan, 1997).

Several studies sought to gain deeper insight into hematopoietic function in Belgrade rats. Homozygous rats display decreased general cellularity in bone marrow than normal rats. For example, early and late erythroid progenitors (BFU-E and CFU-E; Pavlovic-Kentera et al., 1989), granulocyte–monocyte progenitors (Stojanovic et al., 1990), and megakaryocyte progenitors (Rolovic et al., 1991) are significantly diminished. In addition, intensive splenic hematopoiesis has been described in *b/b* rats, indicated by increased iron uptake by spleen, expression of erythroid differentiation markers and elevated erythropoietin serum levels (Pavlovic-Kentera et al., 1989; Biljanovic-Paunovic et al., 1992; Ivanovic, 1997). Indeed, while 42-day-old *+/b* rats have a spleen weight fraction of 0.32 ± 0.1, age- and diet-matched *b/b* rats have a spleen weight fraction of 1.50 ± 0.13 (both values are % body weight; *n* = 3–4; *P* <0.05; J. Kim and M. Wessling-Resnick, personal observations).

### DUODENAL ABSORPTION OF IRON AND MANGANESE

Early studies by Morgan’s group (Farcich and Morgan, 1992a) demonstrated reduced intestinal iron absorption by Belgrade rats, a key finding supported by later work from several groups under different experimental conditions (Knopfel et al., 2005b; Thompson et al., 2007a). Since duodenal iron absorption is highly regulated by iron status, Morgan and colleague also characterized absorption by Belgrade rats fed diets of normal, low, and high iron content (Oates and Morgan, 1996). While duodenal iron uptake in control *+/b* rats varied inversely with iron intake, *b/b* rats failed to show changes under iron loading or iron deficiency. It was later shown that a bolus of dietary iron could induce a rapid decrease in intestinal DMT1 mRNA in *+/b* and *b/b* rats (Yeh et al., 2000). In addition, this study showed that *b/b* rats had generally higher basal protein levels of DMT1. It was also observed that *b/b* rats have lower duodenal DMT1 protein than expected based on the higher mRNA levels, possibly due to impaired release of the mutant protein from its site of synthesis or accelerated degradation (Morgan and Oates, 2002). Finally, TfR gene expression and Tf-bound iron uptake by duodenal enterocytes also has been investigated in the Belgrade rat. TfR1 mRNA expression in *b/b* epithelial cells of the crypt region and crypt–villus junction and ^125^I-labeled Tf uptake in *b/b* villus cells were both similar to Wistar rats, while uptake was significantly greater in *b/b* crypt cells (Oates et al., 2000). These observations suggest that uptake of Tf by enterocytes is largely independent of DMT1’s activity, while it remains unknown if iron delivery by Tf to these intestinal cells might be affected in the Belgrade rat.

Molecular studies on the G185R mutant of DMT1 have shown that transfected cells will target the protein product to the plasma membrane and endosomes although levels are lower than wildtype at the cell surface (Su et al., 1998; Touret et al., 2004). These studies have suggested the mutant has reduced residual activity. Whether the Belgrade rat survives on this reduced activity, or if some other mechanism compensates for iron absorption has been studied (Yeh et al., 2011). Gene expression studies showed HIF-2α expression was increased in *b/b* rats compared with +/+ rats with greater expression in the villus compared to crypt, a response that correlated with the presence of hypoxic protein adducts. Under hypoxic conditions, compounds as nitroimidazole or some of its derivatives, enter viable cells and interact with thiol groups of intracellular proteins thus forming the adducts. Under normal oxygen levels the compounds are reoxidized and diffuse out of the cells.

Moreover, most of the genes whose protein products are responsible for duodenal iron transport were up-regulated. One factor that did not increase was ZIP14, suggesting that it is unlikely to compensate for iron absorption (Yeh et al., 2011). There may be other parallel uptake pathways for iron, however. This idea is supported by studies of neonatal iron assimilation in Belgrade pups. After administration of ^59^Fe to lactating foster dams, total levels of assimilated ^59^Fe between suckling *b/b* and *+/b* pups were not different. However, the examination of blood compartments in *b/b* pups showed elevated iron levels in serum and reduced levels in RBCs compared to *+/b* siblings. Tissue iron distribution was significantly higher in heart, kidney, liver, spleen, and intestine in homozygous pups (Thompson et al., 2007a). Thus, during lactation iron absorption occurs normally, but delivery to red cells is impaired in *b/b* rats causing apparent iron loading in other peripheral tissues. Later on, iron absorption is impaired in adult Belgrade rats as discussed above. Thus, DMT1 does not appear to play a significant role in iron assimilation during lactation, but a developmental transition to DMT1-mediated iron uptake occurs as food intake begins. What factors mediate iron absorption in early development remain to be better characterized, but may provide some clues to additional mechanisms that could compensate for the intestinal uptake defect in the Belgrade rat.

Belgrade rats have also been used to study duodenal transport of manganese and the role of DMT1. Chua and Morgan (1997) evaluated manganese absorption from the intestine using closed *in situ* loops of duodenum and distinguishing between uptake, transfer, and absorption. Manganese uptake was decreased in *b/b* rats compared to *+/b* rats. Although manganese transfer from the duodenum into the carcass was similar in both genotypes, the percentage of absorption in homozygous rats was significantly low. Hence, the primary defect appears to be the absorption step responsible for the uptake of manganese from the gut lumen implicating DMT1 in this process. Indeed, studies from our own laboratory have indicated that Belgrade rats have lower blood manganese levels, supporting the idea that they have manganese as well as iron deficiency. Levels of manganese are 14.6 ± 2.3 versus 8.1 ± 1.2 ng/g blood in *+/b* versus *b/b* rats (±SEM, *n* = 7, *P* = 0.0029; J. Kim and M. Wessling-Resnick, personal observations).

### HEPATIC HANDLING OF IRON AND MANGANESE

**FIGURE 5:**
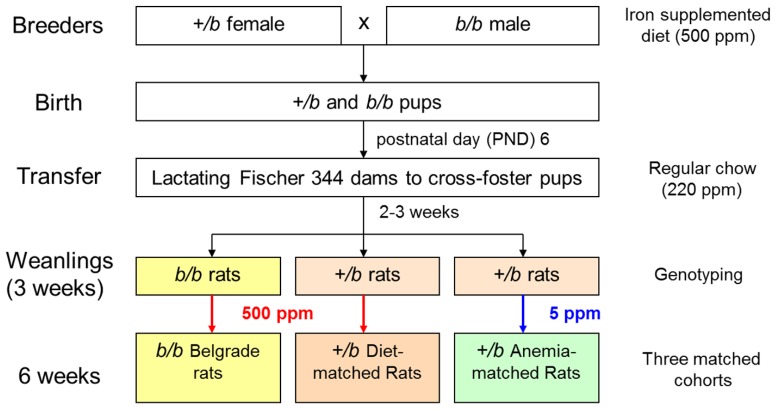
**Breeding and maintenance of the Belgrade rat**.

Iron accumulation in tissues of *b/b* rats after parenteral iron administration was reported early on in the characterization of the Belgrade phenotype (Sladic-Simic et al., 1969). In fact, this feature defect resembled thalassemia, an iron-loading anemia. When the first human mutations in DMT1 were discovered (Mims et al., 2005; Beaumont et al., 2006; Lam-Yuk-Tseung et al., 2006), patients were found to suffer not only from anemia but also from hepatic iron overload. It was suggested that rodent models of DMT1 deficiency did not display iron overload since heme iron was not present in chow, and that the human condition might be caused up-regulation of heme absorption. Our laboratory investigated this issue in the Belgrade rat model (Thompson et al., 2006). First, female *+/b* rats crossed with male *b/b* rats were fed an iron-supplemented diet (500 ppm) to support pregnancy. After birth, litters were cross-fostered to F344 Fischer dams fed a standard diet, and upon weaning both *+/b* and *b/b* pups were fed an iron-supplemented diet 3 weeks. To control for the anemic status of the Belgrade rats, *+/b* rats are fed a low iron diet (5 ppm) under our laboratory’s husbandry protocol (**Figure 5**). Despite the anemic state of *b/b* rats, liver non-heme iron content was greater compared with age-matched (and diet-matched) *+/b* sibling controls. Perl’s Prussian blue staining showed iron deposition was evident in both periportal and centrilobular zones. In contrast, no iron staining was observed in age-matched *+/b* rats (Thompson et al., 2006). This pattern of liver iron deposition suggested that the primary defect in erythron iron utilization seen in homozygous rats leads to liver iron loading. Consistent with this idea, the Belgrade rats also display higher serum iron levels (Kim et al., 2013). These data argue that the liver can acquire iron independent of DMT1, in agreement with previous studies that showed iron-loading in DMT1 knockout mice (Gunshin et al., 2005). The high hepatic expression of Zip14 – which is up-regulated by high iron (Nam et al., 2013) – raises the likelihood that this transporter is responsible. Interestingly, quantitative real-time RT-PCR analysis showed hepcidin expression was threefold higher in *b/b* compared to *+/b* littermates (Thompson et al., 2006), consistent with studies showing that hepcidin expression increases with liver iron loading despite severe anemia (Vokurka et al., 2006). Yeh et al. (2011) have also reported hepcidin expression increases in iron-fed *b/b* rats. Thus, the Belgrade rat’s inability to take up adequate iron might be compounded by hepcidin’s down-regulation of ferroportin, the basolateral iron exporter. Recent studies in mice with the Dmt1 gene selectively inactivated in hepatocytes reinforce the idea that hepatic DMT1 is dispensable for NTBI uptake, although they also showed unaffected hepatic iron levels in these mice, suggesting that DMT1 is also not essential for hepatic iron accumulation (Wang and Knutson, 2013).

Manganese as well as iron content is altered in liver of Belgrade rats. It has been reported that the concentration and total content of manganese in liver is significantly less in *b/b* rats compared to *+/b* rats (Chua and Morgan, 1997). As discussed above, intestinal manganese absorption is diminished in Belgrade rats. Interestingly, a significantly higher uptake of ^54^Mn into liver was observed when it is administered as Mn–Tf or Mn–serum (Chua and Morgan, 1997). The difference between iron and manganese metabolism is that while iron is retained, and therefore might accumulate due to ineffective erythroid uptake, excess manganese is rapidly cleared by the liver (Papavasiliou et al., 1966). The fact that hepatic uptake is increased suggests the liver import mechanisms of these two metals once again overlap: up-regulation of Zip14 or another transporter might induce this effect.

### RENAL IRON HANDLING

Despite the high renal expression of DMT1, we only have a minimal understanding of its function in this tissue. Studies of the renal physiology of Belgrade rats have helped to shed some light. Analysis of renal DMT1 mRNA showed the same transcript size in *b/b* and *+/b* rats, without changes in levels of mRNA expression between genotypes (Ferguson et al., 2003). Expression of DMT1 protein is reduced in the kidneys, but this might be expected since protein levels do not generally reflect transcript levels. Reduced immunostaining for DMT1 was not specific for a region, since it is observed in proximal, distal, and collecting tubules. Once again, these observations indicate that the mutation may accelerate DMT1 degradation or cause defective posttranslational processing (Ferguson et al., 2003).

General tissue disorganization and abnormal morphology of cortical tubules of the Belgrade rat kidney was first noted by Ferguson et al. (2003). A later study from our group described glomerulosclerosis and interstitial sclerosis in *b/b* rats upon aging, with fibrosis in glomeruli and areas of tubulointerstitial fibrosis (Veuthey et al., 2013). Tubular dilation with flattened epithelium in some cortical tubules and occlusion of the luminal space in other cases was observed. Although serum creatinine appears normal in *b/b* rats, creatinine clearance was significantly reduced suggesting a decrease in the glomerular filtration rate. Moreover, elevated urinary albumin in *b/b* compared to *+/b* rats has been reported by us (Veuthey et al., 2013), suggesting damage in the glomerular membrane (Satchell and Tooke, 2008). Since albumin is normally reabsorbed in the tubular system, the evidence suggests that the increased tubular burden of albumin could be associated with progressive interstitial fibrosis and tubular damage (Eddy, 1994; Jerums et al., 1997; Wang and Hirschberg, 2000).

A detailed measurement of several electrolytes has been carried out in feces, serum, and urine of Belgrade rats (Ferguson et al., 2003). Fecal excretion of almost all tested ions was similar between *b/b* and *+/b* animals, except for Fe^2^^+^, which was higher in *b/b* rats. In addition, serum levels of electrolytes showed high Mg^2^^+^ and decreased K^+^ in *b/b* compared to *+/b* rats. Urinary analysis revealed higher Ca^2^^+^ levels but without changes in Fe levels. This last finding differs from studies of the Belgrade rat in our lab that show higher urinary iron output; the age of rats that were studied could be one factor accounting for this difference since kidney function worsens with age in Belgrade rats (Jia et al., 2013; Veuthey et al., 2013). The fact that more iron is excreted in *b/b* rat urine could result from the altered glomerular membrane function, but also from loss of tubular reabsorption. Evidence suggests that both pathways could be affected in *b/b* rats (Veuthey et al., 2013). Since tubular uptake of Fe^3^^+^ bound to Tf seems to be mediated by cubilin, the high urinary Tf seen in *b/b* together with the absence of changes in cubilin suggest that tubular reabsorption of Tf is not affected.

Another interesting finding related to the renal pathology was the premature death of homozygous Belgrade rats. We observed that several early urinary biomarkers of renal injury were altered *b/b* rats, pointing to a kidney defect (Veuthey et al., 2013). The evaluation of early renal development in *b/b* pups by the radial glomerular count (RGC) method supports the idea that limited iron supply during early life could affect renal development in adults leading to injury and even death from renal failure (Drake et al., 2009; Veuthey et al., 2013). Maternal iron restriction during pregnancy has been previously documented to induce altered renal morphology in adult offspring (Lisle et al., 2003). Nephron number is set early in life and does not increase (Al-Awqati and Preisig, 1999). Our RGC study of nephrogenesis indicated that *b/b* pups have decreased nephron allotment. Brenner et al. (1996) have put forward the hyperfiltration hypothesis that these early development defects explain compromised renal metabolism observed in adulthood. Adaptive activity of remnant nephrons would need to maintain glomerular filtration. Over time, increased glomerular pressure promotes fibrosis and sclerosis to produce glomerular injury. Such injury leads to further nephron loss, thereby continuing a vicious cycle that finally decreases the glomerular filtration, ending in renal damage and poor kidney function.

### RESPIRATORY AND OLFACTORY UPTAKE

Due to the nature of its transport and metabolism, airborne manganese promotes neurotoxicity upon its distribution to the brain. Inhalation exposures to manganese pose a significant occupational health risk to welders, for example, who are exposed to fumes containing iron, chromium, manganese, aluminum, nickel, and cadmium. Within these mixtures are multiple transport substrates for DMT1. Our laboratory was interested in the potential role DMT1 played in this process due to the fact this pathway would be up-regulated during iron deficiency. “Iron-responsive manganese uptake” would exacerbate the neurotoxicity of airborne metal. Here, the Belgrade rat was used as a model system, with cohorts of homozygous, heterozygous, and anemic heterozygous controls (**Figure 5**). Uptake of intranasally instilled ^54^Mn was markedly reduced in *b/b* rats compared to iron-replete *+/b* rats (Thompson et al., 2007b). Enhanced ^54^Mn absorption was observed in iron-deficient *+/b* controls relative to both *b/b* and iron-replete +/b rats. In contrast, ^54^Mn clearance from blood to peripheral tissues showed the same pharmacokinetics for intravenously injected *b/b*, *+/b* and iron-deficient *+/b* rats. The sum of this pharmacokinetic data supports a functional role for uptake of DMT1 in manganese uptake by the olfactory pathway. Immunohistochemistry revealed that DMT1 was associated with the microvilli of the olfactory epithelium and the endfeet of olfactory epithelial sustentacular cells. Most importantly, DMT1 levels in the olfactory epithelium were significantly greater in iron-deficient rats. Finally, the fact that total levels of Mn in brain of intranasally instilled rats were 10-fold higher than in intravenous injected animals directly demonstrates that inhalation promotes greater brain manganese uptake. Thus, the apparent function of DMT1 in olfactory Mn absorption suggests that manganese neurotoxicity can be modified by iron status due to the iron-responsive regulation of DMT1. Subsequent studies of the Belgrade rat also showed that lack of DMT1 can affect the absorption of iron from the nasal cavity to the blood and finally to the brain (Ruvin Kumara and Wessling-Resnick, 2012), further localizing iron-regulated DMT1 expression in the olfactory bulb, too.

The Belgrade rat also has been used to investigate whether DMT1 plays a role in uptake across the pulmonary epithelium of the lungs. Previous investigation by others showed the Belgrade rat has reduced clearance of iron (Wang et al., 2002). In our intratracheal instillation experiments, the transport of ^54^Mn to the blood was unaltered (Brain et al., 2006). However, studies of the Belgrade rat do suggest that DMT1 is involved in pulmonary inflammation (Wang et al., 2005; Kim et al., 2011) as well as metal-induced injury (Ghio et al., 2005, 2007), emphasizing an important detoxification pathway that can transport and sequester metals in the lungs after inhalation exposures. Using the Belgrade rat model, Ghio and colleagues demonstrated that ozone-induced lung injury was dependent on DMT1 and that the transporter could modify oxidative stress responses. Lipopolysaccharide (LPS) also has been shown to induce DMT1 (Nguyen et al., 2006), supporting that idea that under infection and inflammation, it functions to take up and sequester metals as a protective host response. The net effect of such alterations would be to reduce levels of manganese available in the lung to limit survival of pathogens like *S. pneumonia* and to help protect the lungs from inflammation due to air pollution and other irritants.

### THE BLOOD–BRAIN BARRIER

Brain metal homeostasis is critical to cognitive development, behavior, and motor control. Excess iron and manganese are also associated with neurodegeneration, for example, in Alzheimer’s and Parkinson’s disease patients. Belgrade rats have been used to study transport of iron and manganese across the blood–brain barrier. Iron content in brain seems to vary depending on the age of *b/b* rats. Similar levels were reported for *b/b* and *+/b* pups, while significantly lower levels were seen in 21-day-old *b/b* rats compared to *+/b* age-matched controls (Thompson et al., 2007a). Burdo et al. (1999) performed a detailed analysis of iron distribution in brain of Belgrade rats. Cortical gray matter showed iron-positive astrocytes in brain of both *b/b* and *+/b* rats, although fewer cells were stained in the Belgrade rat cohort. Iron was also observed in pyramidal neurons, but they were fewer in number and less intensely stained than in *+/b* rats. In white matter of *+/b* rats, iron was present in patches of intensely iron-stained oligodendrocytes and myelin, while the same pattern of expression was seen but with dramatically less intensity in homozygous rats. In addition, the stained oligodendrocytes were associated with blood vessels. The general decrease in iron staining observed in *b/b* rats compared to *+/b* is consistent with the decreased Tf and iron uptake into the brain previously reported for Belgrade rats (Farcich and Morgan, 1992a). Loss of neuronal iron staining in brain of *b/b* agrees with the finding that DMT1 mRNA is mainly expressed in these cells (Gunshin et al., 1997).

Early in 1992, impaired iron uptake by *b/b* rat brain was reported, although at that time the details of this mechanism were not clear (Farcich and Morgan, 1992a). Later on, it was established brain uptake of Tf-bound iron was decreased in young and adult *b/b* rats compared to *+/b* control rats, while Tf uptake was similar between both cohorts (Moos and Morgan, 2004). These authors also reported significantly lower iron content in brain of *b/b* rats together with higher expression of neuronal TfR1, confirming the iron-deficient stage of homozygous rats. By looking at the brain capillary endothelial cells (BCECs) involved in the transport trough the blood–brain barrier, TfR expression appeared to be identical. DMT1 was mainly detected in the cytoplasm of neurons and choroid plexus epithelial cells, but was not detected in BCECs (Moos and Morgan, 2004).

Based in that study, it has been proposed that neurons could acquire iron by receptor-mediated endocytosis of Tf, followed by iron transport out of endosomes mediated by DMT1. In Belgrade rats, the mutation on DMT1 could explain the low cerebral iron uptake, suggesting that it is due to a reduced neuronal uptake rather than an impaired transport trough the blood–brain barrier. For manganese, however, the story is much less clear. Uptake of intravenously injected ^54^Mn into the brain was similar for *b/b* and *+/b* rats, while iron-deficient *+/b* controls took up less. Since presumably both TfR and DMT1 would be up-regulated in the latter cohort, these data suggest the pathway for manganese may be different than iron uptake into the brain. This evidence agrees with previous reports that DMT1 is not involved in the blood–brain transport of manganese (Crossgrove and Yokel, 2004).

### LIPID METABOLISM

Belgrade rats also exhibit pathological changes in lipid metabolism. Homozygous *b/b* rats display hypertriglyceridemia and elevated free fatty acids compared to *+/b* rats, without significant changes in cholesterol levels (Kim et al., 2013). Lipoprotein triglycerides (TGs) are associated with very low density lipoprotein (VLDL). Since hepatic TG levels were similar in *b/b* and *+/b* rats, higher serum TG levels in *b/b* rats appear to be unrelated to increased production, an idea supported by the fact that hepatic lipogenic gene expression is not affected. Instead, all of the evidence points to a block in TG uptake, which can be explained by the fact that serum lipoprotein lipase (LPL) activity is significantly reduced in *b/b* rats compared to *+/b* age-matched controls (Kim et al., 2013). LPL is the key enzyme responsible for release of TG lipids for uptake after VLDL binds to its receptor. The fact that VLDL receptor levels in muscle [and low-density lipoprotein (LDL) receptor levels in liver] are similar in *b/b* and *+/b* rats supports the idea that LPL inhibition accounts for hypertriglyceridemia. The unusual iron-loading anemia of the Belgrade rat suggested an interaction with high serum iron. Indeed, serum LPL activity is also reduced in rats with dietary iron loading, confirming the fact that increased serum iron is associated with decreased LPL activity. Based on these data, our laboratory studied how iron alters serum LPL *ex vivo* or recombinant LPL *in vitro*, finding that exogenously added iron inhibits this enzyme in a dose-dependent manner. These independent lines of evidence suggests that elevated serum iron levels of *b/b* rats promote reduced TG clearance due to LPL inhibition, resulting in higher levels of serum TG. Although the molecular basis for iron-mediated regulation of LPL activity is not clear, oxidative modification of the enzyme, its substrate, and/or reaction products could interfere with lipolysis (Kim et al., 2013). Clinically, reduction of iron loading in patients by phlebotomy (Casanova-Esteban et al., 2011), chelation (Cutler, 1989) and diet (Cooksey et al., 2010) can help to improve lipid disorders. We also found that reducing serum iron in Belgrade rats by treatment with the uptake inhibitor ferristatin II improved their TG levels, suggesting pharmacological interventions could be helpful (Kim et al., 2013).

### GLUCOSE METABOLISM

Despite their hyperferremia, Belgrade rats display normal insulin and glucose tolerance (Jia et al., 2013). Comparable levels of insulin-induced Akt phosphorylation in *b/b* and *+/b* rats suggests that downstream insulin signaling is also unaffected. Moreover, insulin secretory capacity of the pancreas showed no significant differences between *b/b* and *+/b* rats, although pancreatic non-heme iron levels were >5-fold higher in Belgrade rats. Histological evaluation suggests an absence of tissue damage despite the known association between high serum iron and tissue damage (Inoue et al., 1997). The sum of these data support that loss of DMT1 protects pancreatic β-cells and helps to maintain insulin sensitivity despite iron overload (Jia et al., 2013). These results correlate very well with studies of the β-cell specific *Dmt1* knockout mouse, which is resistant to diabetes (Hansen et al., 2012).

An unusual characteristic of Belgrade rats is that they consume more food that heterozygous littermates but display lower body weight, suggesting an apparent imbalance in energy metabolism (Ferguson et al., 2003; Jia et al., 2013). That unexpected phenotype has been associated, at least in part, with increased urinary glucose excretion. Since the kidneys play an important role in glucose homeostasis through tubular reabsorption, the glycosuria observed could be explained by the abnormalities reported in kidney histology and physiology discussed above (Jia et al., 2013; Veuthey et al., 2013). Collectively, these surprising findings reveal that lack of DMT1 function could have an unexpected and significant role in energy balance.

### TRANSPORT OF OTHER METALS

As its name implies, DMT1 not only services iron and manganese metabolism, but it is also involved in uptake of other metals. Knopfel et al. (2005b) isolated brush border membrane (BBM) vesicles to study nickel transport in Belgrade rats. This group measured active nickel transport in BBM vesicles from *+/b* rats but not *b/b* rats. This result implies that the Belgrade mutation disables the transport capacity for this metal. The potential role of DMT1 in copper transport also has been evaluated using the same approach. When unenergized vesicles were utilized, transport of copper was disrupted in BBM vesicles isolated from *b/b* rats, while *+/b* vesicles showed normal copper transport (Knopfel et al., 2005a). Interestingly, when ATP-loaded or energized vesicles were studied, transport characteristics for this metal appeared to be identical in *b/b* and *+/b* rats. It was suggested that ATP-driven copper uptake is a principal copper transport mechanism, while DMT1 might act under conditions of copper excess. Since Belgrade rats are not copper deficient despite DMT1 mutation and iron deficiency, this evidence agrees with previous reports suggesting the existence of several copper transporters other than DMT1 in the intestinal BBM (Knopfel et al., 2005a).

However, the influence of copper in iron metabolism, and reciprocally, the influence of iron status on copper metabolism, presents an interesting and compelling relationship (Yokoi et al., 1991; Ece et al., 1997). Induction of the duodenal Menkes copper ATPase (Atp7a) and metallothionein (Mt1a) have been described in iron-deficient rats, together with high serum and hepatic copper levels and increased ceruloplasmin (Collins et al., 2005). Whether or not DMT1 could contribute to copper uptake remains controversial, with evidence for (Arredondo et al., 2003; Espinoza et al., 2012) and against (Illing et al., 2012; Shawki et al., 2012). Moreover, the valency of copper as a potential transport substrate (Cu^+^ versus Cu^2^^+^) must be considered. Jiang et al. (2013) addressed some of these questions using the Belgrade rat model by comparing *b/b* rats with diet-matched *+/b* and anemic *+/b* littermate controls (**Figure 5**, for example). Previous work by this group showed Belgrade rats did not display the expected changes in hepatic or serum copper upon iron deficiency, suggesting DMT1 might play a role in these processes (Jiang et al., 2011). To test this more directly, an everted gut sac transport assay was used to assess Cu^+^ transport. Increased uptake in iron-deficient *+/b* control rats was observed while *b/b* and diet-matched *+/b* controls had similar levels of uptake. Thus, DMT1 could play a role in copper uptake under iron-deficiency conditions.

Interestingly, DMT1 demonstrates a stronger selectivity for Cd^2^^+^ than Fe^2^^+^ or other more physiologically relevant metals (Illing et al., 2012). This toxic metal is absorbed by the intestine and other tissues and can exert nephrotoxicity. The notion that DMT1 might play a role in Cd distribution and uptake remains to be tested – and the Belgrade rat may provide an excellent model system to explore this possibility.

## CONCLUSION

Although murine models are perhaps more often used to study mammalian metabolism, rat models have provided critical information about obesity (Zucker fatty rat), diabetes (Wistar fatty rat; Otsuka Long–Evans Tokushima fatty rats; Goto-Kakizaki rat); hypertension (Spontaneously hypertensive rat; Dahl salt-sensitive rat), copper metabolism (Long–Evans cinnamon rat), and renal metabolism (ZSF1 rat). We have outlined multiple examples where the Belgrade rat, as a model of iron deficiency, has been useful in characterizing not only the role of DMT1 in transport of this metal, but also its contribution to pathologies of intermediary metabolism, its protective role in detoxification of the lungs, its participation in neurotoxicity of airborne metal uptake by the olfactory pathway, in the development of the kidneys, in promoting altered renal function, in brain iron metabolism and in hepatic iron handling. While the Belgrade rat is the first known rat model of inherited anemia, recently Bartnikas et al. (2013) described a rat model of hereditary hemochromatosis. This group suggests that rats homozygous for a novel Ala679Gly allele of the TfR2 gene spontaneously load iron, thus recapitulating the human disease. Although many murine models of hemochromatosis exist, none exhibit fibrosis of the liver, a debilitating aspect in human patients with this disease. This opens the exciting possibility to explore new metabolic features associated with inherited iron overload with the potential to explore therapeutic avenues that will ameliorate the disease. Moreover, as genetic manipulation of the rat becomes more routinely available, future models may contribute a better understanding of the genes of iron metabolism – in both iron deficiency and overload – and their contributions to mammalian physiology and pathology.

## Conflict of Interest Statement

The authors declare that the research was conducted in the absence of any commercial or financial relationships that could be construed as a potential conflict of interest.
